# Tetramethylpyrazine Enhances Vascularization and Prevents Osteonecrosis in Steroid-Treated Rats

**DOI:** 10.1155/2015/315850

**Published:** 2015-02-11

**Authors:** Yini Jiang, Chunfang Liu, Weiheng Chen, Hui Wang, Chao Wang, Na Lin

**Affiliations:** ^1^Institute of Chinese Materia Medica, China Academy of Chinese Medical Sciences, No. 16, Nanxiaojie, Dongzhimennei, Beijing 100700, China; ^2^School of Pharmacy, Jiangxi University of Traditional Chinese Medicine, Nanchang 330004, China; ^3^Wangjing Hospital, China Academy of Chinese Medical Sciences, Beijing 100102, China

## Abstract

Steroid-induced osteonecrosis of the femoral head (steroid-induced ONFH) is an avascular necrosis disease of bone. Tetramethylpyrazine (TMP), with significant vascular protective properties, has been widely used for the treatments of ischemic neural disorders and cardiovascular diseases. However, its role in the treatment of steroid-induced ONFH has not been evaluated. In this study, our results showed that TMP significantly decreased the ratio of empty lacuna, adipose tissue area, and adipocyte perimeter in steroid-induced ONFH rats histopathologically. TMP also reduced the levels of serum lipid dramatically by haematological examination. According to the micro-CT quantification, TMP could improve the microstructure of the trabecular bone and increases bone mineral density in steroid-induced ONFH rats. Moreover, TMP significantly increased the vessel volume, vessel surface, percentage of vessel volume, and vessel thickness of the femoral heads by micro-CT. Interestingly, the downregulation of VEGF and FLK1 proteins in the sera and necrotic femoral heads could be reversed by TMP treatment, and this was true for their mRNA expressions in femoral heads. In conclusion, these findings suggest for the first time that TMP may prevent steroid-induced ONFH and also enhance femoral head vascularization by inhibiting the effect of steroid on VEGF/FLK1 signal pathway.

## 1. Introduction

Steroid-induced osteonecrosis of the femoral head (steroid-induced ONFH) represents a degenerative bone disease characterized by the increase of intraosseous pressure because of lipid metabolism disturbance such as elevating of adipogenesis and fat cell hypertrophy in the bone marrow, subsequently leading to disturbances of coagulation-fibrinolysis system in the femoral head and finally resulting in bone ischemia [1]. It is a common complication with disabling effect for patients after high-dose corticosteroid treatment [2]. Previous studies have demonstrated that steroids decrease skeletal angiogenesis, vascularity, and hydration [3], reduce femoral head blood flow, and have a vasoconstrictive effect on lateral epiphyseal arteries of the femoral head which could lead to femoral head ischemia and subsequent necrosis [4, 5]. The pathogenesis and aetiology of steroid-induced ONFH have not been fully elucidated. According to the recent study, increased osteocytes apoptosis, the impairment of bone cell survival and bone formation, promotion of osteoclastic resorption, and adipocytic differentiation in bone microenvironments as well as ischemia may be the assumed causes for this disease [6, 7]. Among these, ischemia is a feature of the necrotic lesion [8]. The current surgical treatments for this disease aimed at hip preservation have attempted to induce revascularization or transposition of the necrotic area [9–11]. However, these treatments were limited in their ability to support subchondral bone and were not necessarily successful. Therefore, it is extremely necessary to develop novel and efficient agents for the treatment of this disease.

Tetramethylpyrazine (TMP, relative molecular mass: 208.5) is one of the most important active ingredients of the traditional Chinese herbal medicine,* Ligusticum wallichii* Franchat (Chuan Xiong) [12, 13]. It has been widely used, especially in the treatments of ischemic neural disorders and cardiovascular diseases, such as ischemic stroke and pulmonary hypertension secondary to chronic obstructive pulmonary diseases, for a long time in China [14–16]. TMP has been demonstrated to inhibit platelet aggregation, dilate blood vessels, depress blood viscosity, improve microcirculation, increase coronary and cerebral blood flow, promote vascular recanalization and endotheliocyte proliferation and migration, and scavenge reactive oxygen species [14, 17–22]. On this basis, we hypothesize that TMP might possess a therapeutic effect on steroid-induced ONFH, an ischemic necrosis disease of bone. Thus, the aim of the current study was to verify whether TMP possesses any preventive effects on the osteonecrotic changes, repair, and revascularization processes in steroid-induced ONFH rats. Moreover, the underlying pharmacological mechanisms of revascularization are investigated.

## 2. Materials and Methods

The study was approved by Research Ethics Committee of China Academy of Chinese Medical Sciences, in accordance with the National Institutes of Health Guidelines for the Care and Use of Laboratory Animals. All animals were treated in accordance with the guidelines and regulations for the use and care of animals of the Center for Laboratory Animal Care, China Academy of Chinese Medical Sciences.

### 2.1. Animals

Eighty-five 12-week-old male Wistar rats (Cat. number SCXK-(Jun) 2007-004) weighting 300–320 g were obtained from Experimental Animal Centre of Academy of Military Medical Sciences (Beijing, China). All rats were maintained in a room equipped with an air-filtering system, and the cages and water were sterilized.

### 2.2. Groups and Treatment

After one week feeding adaptation, the animals were accurately weighed and randomly divided into four groups: control (*n* = 20), model (steroid-induced ONFH rats, *n* = 25), TMP 20 mg/kg (steroid-induced ONFH rats treated with 20 mg/kg TMP, *n* = 20), and TMP 40 mg/kg (steroid-induced ONFH rats treated with 40 mg/kg TMP, *n* = 20). Steroid-induced ONFH rat models were constructed according to the previous studies [23, 24]. Briefly, rats were injected subcutaneously at the back by methylprednisolone acetate (MPSL; Pfizer Manufacturing BeLgium NV, Puurs, Belgium) (21 mg/kg) daily for six weeks to induce osteonecrosis. One hour after the MPSL injection per day, rats in TMP 20 mg/kg and TMP 40 mg/kg groups, respectively, received ligustrazine hydrochloride (Tianjin Pharmaceutical Jiaozuo Co., Ltd., Jiaozuo, China) by intramuscular injection at the hip with the dosages of 20 mg/kg/day and 40 mg/kg/day for six weeks. In the control and model groups, the rats received distilled water. The animals were fed a standard diet and allowed free activity.

### 2.3. Tissue Sample Preparation

Rats from each group were killed six weeks after the MPSL injection. The rats were anaesthetized with an intravenous injection of trichloroacetaldehyde hydrate (0.3 mL/kg, Sinopharm Chemical Reagent Co., Ltd, China) and were then killed by exsanguination via an aorta. The left sides of femora were obtained and fixed for three days in 4% paraformaldehyde (pH 7.4) and then decalcified with 10% EDTA for one month. Samples were sectioned along the coronal plane for the proximal one-third and cut along the axial plane in the distal part (condyle). Finally, the specimens were embedded in paraffin, cut into 5 *μ*m sections, and stained with haematoxylin and eosin. The right sides were stored at −80°C for western blot and real-time PCR test.

### 2.4. Evaluation of Steroid-Induced ONFH

The osteonecrotic changes and repair processes in steroid-treated rats were observed by the histopathological examination using a light microscope six weeks after the MPSL injection. The slides were evaluated in a blinded fashion by three independent observers. The evaluation criteria for osteonecrosis were based on the report of Yamamoto et al. [25]. Osteonecrosis was judged to be present when there was necrosis of medullary hematopoietic cells or fat cells or there were empty lacunae or condensed nuclei in osteocytes. The ratio of empty lacunae (empty lacunae/the total number of osteocytes) was calculated for each femoral head using a coronal section taken at the maximal femoral width. A computerized image analysis program (image pro 6.0) was used for this calculation.

### 2.5. Micro-CT

A micro-CT (GE Healthcare Biosciences, Piscataway, NJ, USA) was used to detect the change of the excised femoral head samples. The following parameters were calculated: bone volume (BV), tissue volume (TV), trabecular bone pattern factor (Tb.Pf), structure model index (SMI), trabecular thickness (Tb.Th), trabecular number (Tb.N), trabecular separation (Tb.Sp), and bone mineral density (BMD). After micro-CT, the decalcified samples were embedded in paraffin and cut into 5 *μ*m sections along the coronal plane for the proximal parts. Sections were stained with hematoxylin and eosin (H&E) for evaluation of ON and repair process.

### 2.6. Quantification and Three-Dimensional Visualization of Vessel Networks

Femoral head blood vascularization in steroid-treated rats was measured using the micro-CT-based microangiography six weeks after the MPSL injection according to the previous studies [26, 27]. Briefly, rats from each group (*n* = 10) were anaesthetized as described above and the thoracic and abdomen cavities of the animals were opened. A hypodermic needle with disposable infusion device was inserted in the ventriculus sinister with ligation of that proximal to the aorta ascendens. The vasculature was flushed with 500 mL 50 U/mL heparinized saline at 37°C and at maximum flow via a disposable infusion device. As soon as the outflow from an incision of the auricula dextra was limpidness, 500 mL 4% paraformaldehyde solution was pumped into the vasculature to fix the tissues and blood vessels. The vasculature was then injected with Microfil based on the manufacturer's protocol (Microfil MV-122, Flow Tech; Carver, MA, USA). Animals were then stored overnight at 4°C to ensure polymerization of the contrast agent before microangiography. Bilateral femoral samples were then harvested, fixed in 4% paraformaldehyde, and decalcified in 10% EDTA. After that, the femoral shaft was fixed in a polymethylmethacrylate sample tube with its long axis perpendicular to the bottom of the tube in preparation for micro-CT scanning. The scan was perpendicular to the shaft and was initiated from a reference line 10 mm away from the bottom with an entire scan length of 10 mm.

### 2.7. Hematological Examination

In order to detect the hyperlipidemia-improving effects of TMP, the blood samples were collected from the abdominal aorta, six weeks after the MPSL injection. The serum levels of total cholesterol (TC), triglycerides (TG), low-density lipoprotein (LDL), high-density lipoprotein (HDL), apolipoprotein A1 (ApoA1), and apolipoprotein B (ApoB) were determined by automatic biochemical analyzer (AU 1000, Olympus, Japan).

### 2.8. Immunohistochemical Staining

Paraffin sections (5 *μ*m) of tissue from the femoral head tissues were mounted on poly-L-lysine-coated slides. The paraffin sections were dewaxed by routine method and incubated for 10 min with 3% H_2_O_2_. The sections were placed in a 37°C, 0.1% trypsinase for 5–30 min for antigen retrieval. Each section was incubated with normal goat serum for 20 min at room temperature and then with primary antibody against rat VEGF (rabbit antibody, dilution 1 : 50, Abcam, Cambridge, MA, UK), FLK1 (rabbit antibody, dilution 1 : 50, Cell Signaling, Boston, USA) overnight at 4°C. After incubation with Polymer Helper for 20 min at 37°C, sections were reacted with poly-HRP anti-rabbit IgG for 20 min at 37°C. The sections were then stained with 3,3-diaminobenzidine (Sigma, St. Louis, MO, USA) and counterstained with hematoxylin. Specimens were examined using a Leica image analyzer and analyzed by computer image analysis (Leica Microsystem Wetzlar Gmbh., Wetzlar, Germany) in a blinded manner. To localize and identify areas with positively stained cells, 10 random digital images per specimen of the femoral head tissues were recorded, and quantitative analysis was performed according to the color cell separation. The results are expressed as the mean region of interest, representing the percentage of area covered with positively stained cells per image at a magnification of 400x.

### 2.9. RNA Isolation and Real-Time PCR

The whole femoral head tissues were dissected from rats six weeks after the MPSL injection, snap-frozen in liquid nitrogen, ground into powder, and homogenized. The RNA isolation and real-time PCR assay were carried out following the protocol of our previous study [28]. Briefly, total RNA was extracted with TRIzol reagent (Invitrogen, Carlsbad, CA, USA) from the tissue homogenates according to the manufacturer's instructions. The total RNA (3 mg) was reverse-transcribed to cDNA using the QuantiTect Reverse Transcription Kit (Thermo Fisher Scientific Inc., CA, USA) according to the instruction manual. The specific transcripts were quantified by quantitative real-time PCR using QuantiTect SYBR Green PCR Kit (Takara Bio Inc., Tokyo, Japan) and analyzed with ABI 7500 real-time PCR system (Applied Biosystems, Foster city, CA, USA). Gene-specific primers were used for VEGF (5′-TTCATGGATGTCTATC AGCG-3′ as forward and 5′-GCTCATCTCTCCTATGTGCT-3′ as reverse), FLK1 (5′-TAGTATT CCGTTCGCAAG-3′ as forward and 5′-TACAACTTTCTCCCTC GT-3′ as reverse), and *β*-actin (5′-ACCCTAAGGCCAACCGTGAAAAG-3′ as forward and 5′-CATGAGGTAGTCTGTCAGGT-3′ as reverse). The mRNA levels of VEGF and FLK1 were normalized to *β*-actin mRNA level. PCR was performed as 40 cycles at 94°C for 15 s, 55°C for 30 s, and 72°C for 30 s. The relative mRNA expression was calculated with comparative CT method.

### 2.10. Western Blot Analysis

The protein expression levels of VEGF, FLK1, and GAPDH in the femoral head tissues obtained from rats in different groups were detected by western blot analysis. The protocol and semiquantitative analysis were carried out following the protocol of our previous study [26]. The following antibodies were used: VEGF antibody (rabbit antibody, dilution 1 : 50, Santa Cruz Biotechnology, Inc., Santa Cruz, CA, USA), FLK1 (rabbit antibody, dilution 1 : 50, Santa Cruz), and GAPDH antibody (internal control, rabbit polyclonal antibody, dilution 1 : 200, Santa Cruz).

### 2.11. Statistical Analysis

The software of SPSS version 13.0 for Windows (SPSS Inc., Chicago, IL, USA) was used for statistical analysis. The results were expressed as $\overset{-}{X}\pm s$. For comparisons of means among multiple groups, one-way ANOVA followed by LSD test was performed. Differences were considered statistically significant when *P* < 0.05.

## 3. Results

### 3.1. TMP Prevents Histopathological Changes in Steroid-Induced ONFH Rats

**Figure 1:**
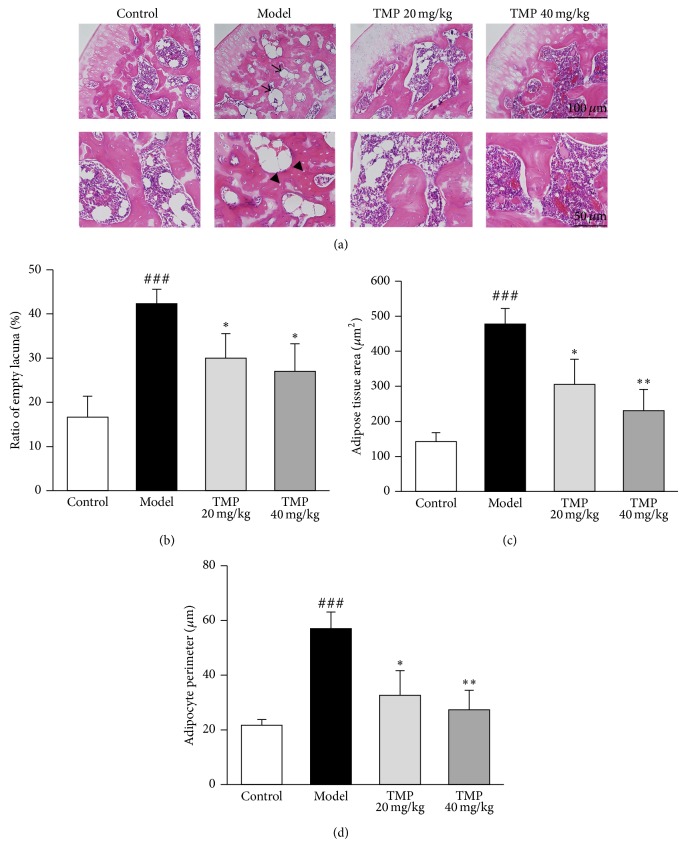
Tetramethylpyrazine (TMP) enhances osteogenesis and reverses bone marrow adipogenesis. (a) Histological features of the normal bone in normal rat and the osteonecrotic bone in steroid-induced ONFH rats with or without TMP administration. Statistical analysis on the differences of the ratio of empty lacuna (b), the adipose tissue area (c), and the adipocyte perimeter (d) in the control, model, TMP 20 mg/kg, and TMP 40 mg/kg groups. Data are represented as the mean ± SD (*n* = 20 for control; *n* = 25 for model; *n* = 20 for TMP 20 mg/kg and 40 mg/kg, resp.). ^###^ *P* < 0.001, compared with the control group. ^*^ *P* < 0.05 and ^**^ *P* < 0.01, respectively, compared with the model group. The arrow heads indicate adipose tissue and the arrows indicate empty lacuna.

The osteonecrotic changes and repair processes of rats in each group were histopathologically observed to evaluate the effect of TMP treatment on steroid-induced ONFH. Compared with the control group, there was an accumulation of bone marrow cell debris found in ONFH lesions in the model group, while TMP treatment could dramatically attenuate this change in steroid-induced ONFH rats (Figure 1(a)). In addition, the ratio of empty lacunae in the bone trabeculae of the model group was significantly more than that of control group but was decreased by the treatment of TMP (Figure 1(b)). Moreover, the adipose tissue area and adipocyte perimeter in the bone marrow, which were dramatically increased in steroid-induced ONFH rats, were both significantly reduced by the TMP treatment (Figures 1(c) and 1(d)). The incidence of osteonecrosis was 23/25 in the model group (92%), 13/20 (65%) in the TMP 20 mg/kg group, and 9/20 (45%) in the TMP 40 mg/kg group, while no osteonecrosis was observed in the control group. Next, apparent adverse effects including weight loss, alterations of physical appearance, and behavior changes were not noted in rats treated with TMP (results not shown). Taken together, these observations indicate that systemic administration of TMP in rats prevents steroid-induced ONFH without serious side effects.

### 3.2. TMP Suppresses Hyperlipidemia in Steroid-Induced ONFH Rats

**Figure 2:**
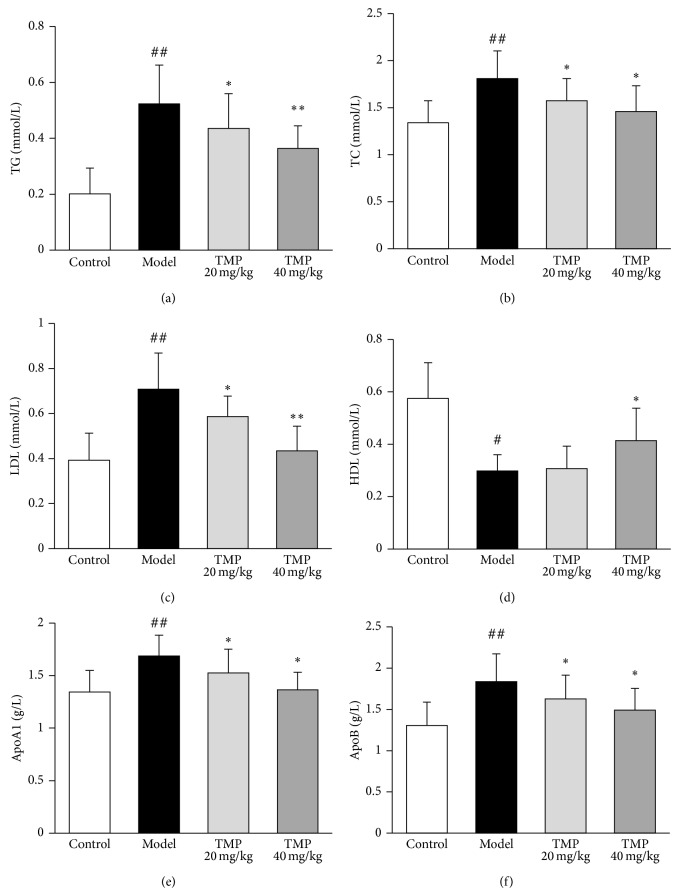
Tetramethylpyrazine (TMP) improves hyperlipidemia in steroid-induced ONFH rats. The steroid hormone administration (model group) induced marked hyperlipidemia, such as significantly elevated TG (a), TC (b), LDL (c), ApoA1 (e), and ApoB (f) levels, but significantly decreased HDL levels (d). More interestingly, doses of 20~40 mg/kg TMP could significantly reduce hyperlipidemia by decreasing TG (a), TC (b), LDL (c), ApoA1 (e), and ApoB (f) levels and increasing HDL levels (d). Data are represented as the mean ± S.D. (*n* = 10 for control; *n* = 15 for model; *n* = 10 for TMP 20 mg/kg, TMP 40 mg/kg, resp.). ^#^ *P* < 0.05 and ^##^ *P* < 0.01, respectively, compared with the control group. ^*^ *P* < 0.05 and ^**^ *P* < 0.01, respectively, compared with the model group.

In the lipid system, the blood chemistry data showed that steroid hormone administration (model group) induced marked hyperlipidemia, such as significantly elevated TG (Figure 2(a)), TC (Figure 2(b)), LDL (Figure 2(c)), ApoA1 (Figure 2(e)), and ApoB (Figure 2(f)) levels, but significantly decreased HDL levels (Figure 2(d)). More interestingly, doses of 20~40 mg/kg TMP could improve hyperlipidemia by decreasing TG (Figure 2(a)), TC (Figure 2(b)), LDL (Figure 2(c)), ApoA1 (Figure 2(e)), and ApoB (Figure 2(f)) levels and increasing HDL levels (Figure 2(d)).

### 3.3. TMP Inhibits the Microstructure Destruction of the Trabecular Bone and Increases BMD in Steroid-Induced ONFH Rats

**Figure 3:**
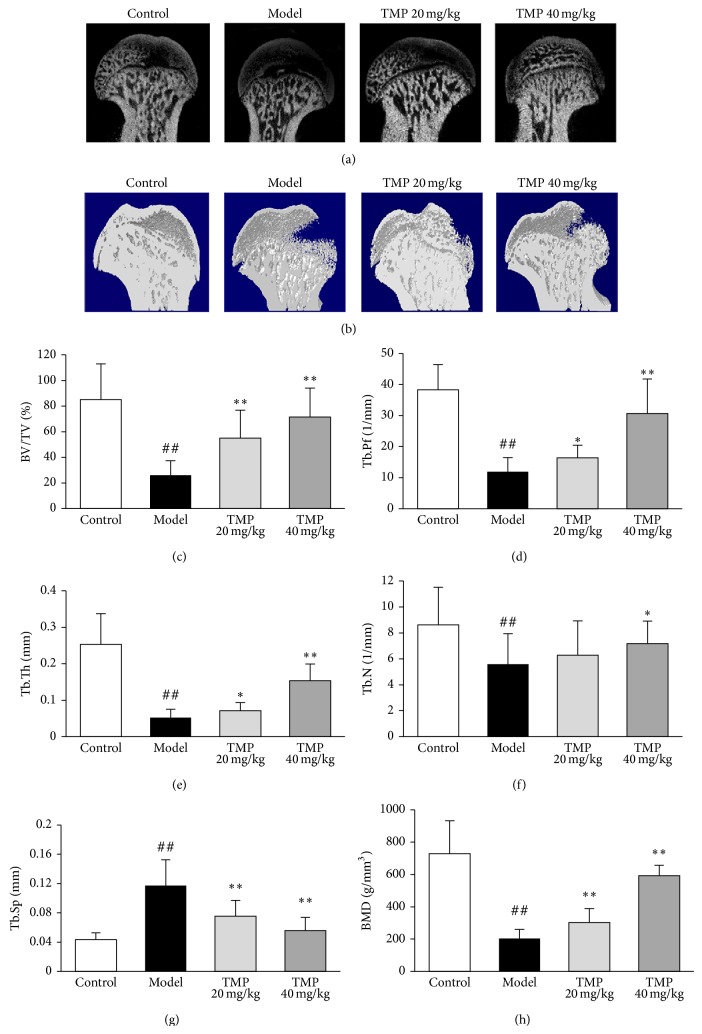
Tetramethylpyrazine (TMP) inhibits the microstructure destruction of the femoral head. (a) and (b), respectively, refer to two-dimensional and three-dimensional pictures of the normal bone in normal rats and the osteonecrotic bone in steroid-induced ONFH rats with or without TMP treatment. Statistical analysis on the differences of the BV/TV (c), Tb.Pf (d), Tb.Th (e), Tb.N (f), Tb.sp (g), and BMD (h) in the control, model, TMP 20 mg/kg, and TMP 40 mg/kg groups. Data are represented as the mean ± S.D. (*n* = 10 for control; *n* = 15 for model; *n* = 10 for TMP 20 mg/kg and TMP 40 mg/kg, resp.). ^##^ *P* < 0.01, compared with the control group. ^*^ *P* < 0.05 and ^**^ *P* < 0.01, respectively, compared with the model group.

Micro-CT scan was performed to validate the efficiency of TMP in steroid-induced ONFH rats. Two-dimensional and three-dimensional reconstruction images from the micro-CT scanning data revealed the degree of microstructure destruction of femoral head in different groups. As shown in Figures 3(a) and 3(b), compared with model group, doses of 20 mg/kg and 40 mg/kg TMP both markedly reduced the extent of microstructure destruction of femoral head. As shown in Figures 3(c)–3(g), microstructural parameters BV/TV, Tb.Th, Tb.Pf, and Tb.N were significantly reduced, while Tb.Sp and SMI were significantly increased in rats with steroid-induced ONFH when compared with controls. TMP treatment protected rats from steroid-induced effects on the levels of the above microstructural parameters. To determine whether TMP treatment increases the bone mass of rats with steroid-induced ONFH, BMD values were calculated. As shown in Figure 3(h), the rats with steroid-induced ONFH showed markedly reduced BMD in the femoral head compared with the control rats. The BMD values in the rats treated with 20–40 mg/kg TMP were increased in a dose-dependent manner compared to those without drug treatment.

### 3.4. TMP Enhances Femoral Head Vascularization in Steroid-Induced ONFH Rats

**Figure 4:**
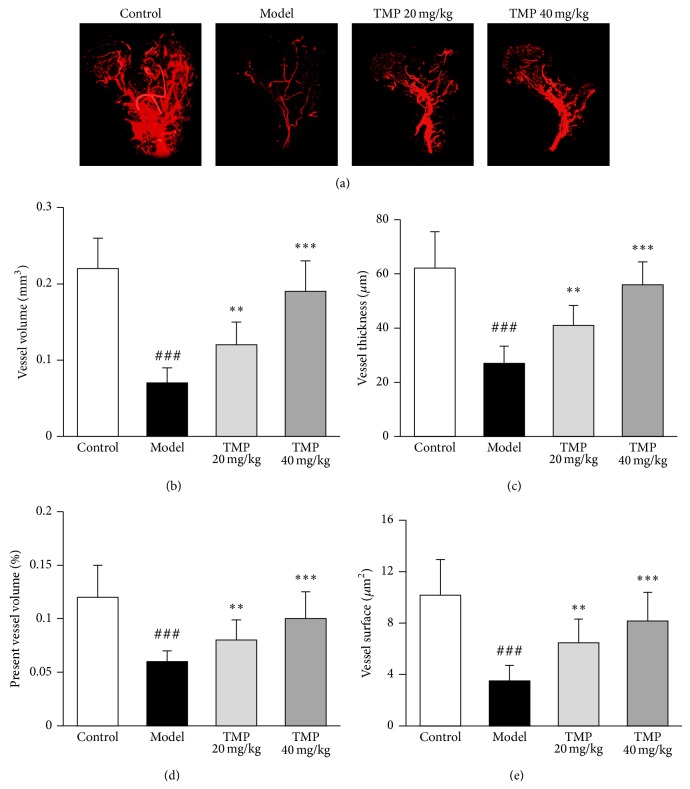
Tetramethylpyrazine (TMP) enhances femoral head neovascularization. (a) Representative images of micro-CT reconstructed 3-dimensional microangiography of proximal femur from control, model, TMP 20 mg/kg, and TMP 40 mg/kg groups. Statistical analysis on the differences of vessel volume (b), vessel thickness (c), percentage of vessel volume (d), and vessel surface (e) in the femoral heads of steroid-induced ONFH rats in different groups. Data are represented as the mean ± S.D. (*n* = 10). ^###^ *P* < 0.001, compared with the control group. ^**^ *P* < 0.01 and ^***^ *P* < 0.001, respectively, compared with the model group.

The blood vessel microarchitecture of each group was reconstructed in three dimensions for presentation. Compared with the control, both the number and the thickness of vessels in the necrotic lesion of femoral head of steroid-induced ONFH rats were markedly reduced and the vasculatures were not visible while the sample in the TMP 20 mg/kg group showed lightly increasing capillary vessels and the sample in the TMP 40 mg/kg groups showed intensive vascular architecture (Figure 4(a)). Quantitatively, TMP increased vessel volume (Figure 4(b)), vessel thickness (Figure 4(c)), percentage of vessel volume (Figure 4(d)), and vessel surface (Figure 4(e)) of the femoral heads of steroid-induced ONFH rats.

### 3.5. TMP Inhibits the Effect of Steroid on VEGF/FLK1 Signal Pathway in the Sera and Femoral Heads of Steroid-Induced ONFH Rats

**Figure 5:**
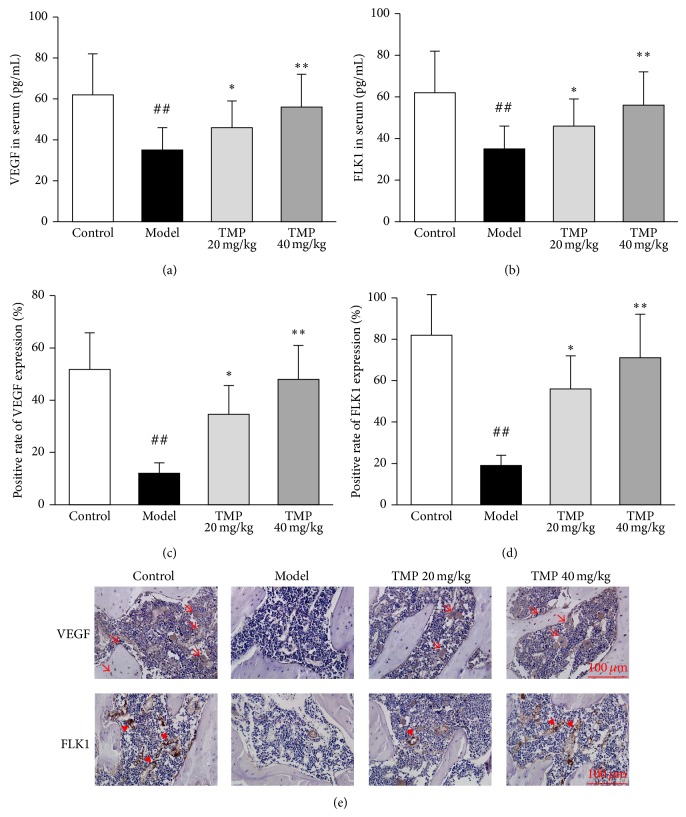
Tetramethylpyrazine (TMP) upregulates the VEGF and FLK1 expression in the sera and femoral heads of steroid-induced ONFH rats by ELISA and immnohistochemistry. The protein levels of VEGF (a) and FLK1 (b) in the sera of steroid-induced ONFH rats in the different groups were detected by ELISA. Statistical analysis of immunohistochemistry on the positive rate of VEGF (c) and FLK1 (d) expression in the femoral heads of steroid-induced ONFH rats from different groups. The positive expressions of VEGF and FLK1 in the femoral heads of steroid-induced ONFH rats from control, model TMP 20 mg/kg, and TMP 40 mg/kg groups were detected by immnohistochemistry (e). The arrows and arrow heads indicate VEGF and FLK1 positive expression cells, respectively. Data are represented as the mean ± S.D. (*n* = 10 for control; *n* = 15 for model; *n* = 10 for TMP treatment groups, resp.). ^##^ *P* < 0.01, compared with the control group. ^*^ *P* < 0.05 and ^**^ *P* < 0.01, respectively, compared with the model group.

**Figure 6:**
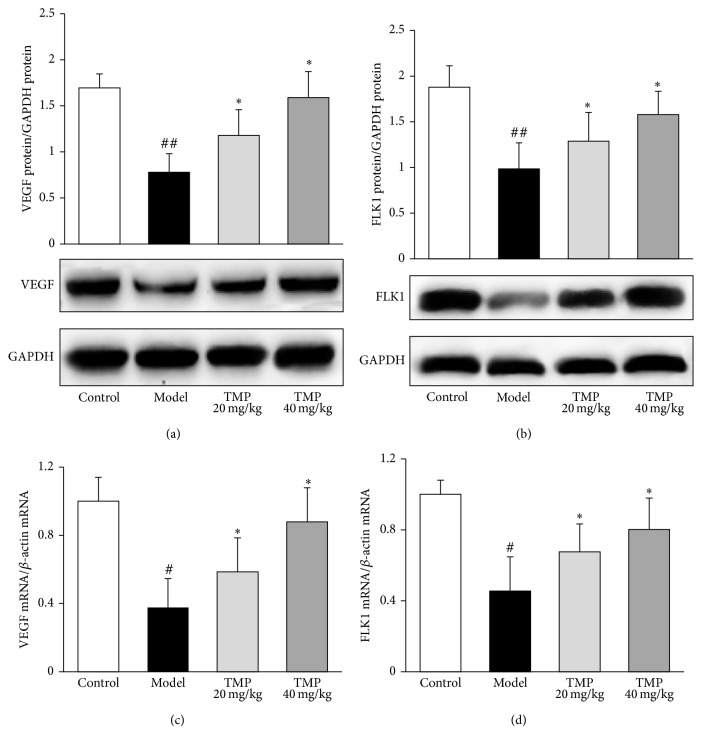
Tetramethylpyrazine (TMP) upregulates VEGF and FLK1 protein and gene expression in the femoral heads of steroid-induced ONFH rats by western blot and real-time RT-PCR. VEGF and FLK1 expressions at both protein and mRNA levels in the femoral heads of steroid-induced ONFH rats with or without TMP treatment were detected by western blot ((a) and (b)) and quantitative real-time RT-PCR ((c) and (d)), respectively. Data are represented as the mean ± S.D. (*n* = 10 for control; *n* = 15 for model; *n* = 10 for TMP treatment groups, resp.). ^#^ *P* < 0.05, and ^##^ *P* < 0.01, compared with the control group, respectively. ^*^ *P* < 0.05, compared with the model group.

We detected the changes of VEGF and FLK1 expression at both protein and mRNA levels in the sera and femoral heads of steroid-induced ONFH rats with or without TMP treatment. As shown in Figures 5(a) and 5(b), the serum VEGF and FLK1 levels in steroid-induced ONFH rats were significantly lower than those in control rats. After the TMP treatment, the serum VEGF and FLK1 levels were dramatically elevated as compared to the model. As shown in Figures 5(c)–5(e), light amounts of VEGF positive staining were present in endothelial cells, osteoblasts, osteocytes and bone marrow haematopoietic cells, and FLK1 positive staining in endothelial cells of the femoral heads from model rats, while there was evident rise of VEGF and FLK1 in the femoral heads from TMP-treated rats. In addition, TMP significantly increased the protein expression of VEGF and FLK1, which were downregulated in the femoral heads of steroid-induced ONFH rats (Figures 6(a) and 6(b)). In line with the findings of western blot analysis, VEGF and FLK1 mRNA expression levels detected by quantitative real-time RT-PCR were also reversed by doses of 20~40 mg/kg TMP treatments significantly (Figures 6(c) and 6(d)).

## 4. Discussion

Steroid treatment is one of the most comment risk factors associated with osteonecrosis [6]. Prolonged steroid use produces a hyperlipidemic state in most patients and subsequently results in abnormal coagulopathy and bone marrow fat-cell packing, leading to microvascular occlusion and high intraosseous pressure, all of which put them at risk for osteonecrosis [29]. Therefore, many researchers postulate that a microvascular enhancing agent may prevent the conditions associated with the development of osteonecrosis. TMP has increasingly gained attention due to its significant vascular protective properties. A number of studies on the effects of TMP on ischemic neural disorders and cardiovascular diseases have been documented [14–16]. However, its role in the treatment of steroid-induced ONFH has not been evaluated. In the current study, we investigate the effect of TMP on steroid-induced ONFH, and the main findings are as the following four points: (1) TMP prevents steroid-induced ONFH by reducing the osteonecrotic changes and the bone marrow adipogenesis of steroid-induced ONFH rats; (2) TMP inhibits the microstructure destruction of the trabecular bone and increases BMD in the femoral head of steroid-induced ONFH rats; (3) TMP enhances femoral head neovascularization and improves hyperlipidemic state of steroid-induced ONFH rats; (4) TMP inhibits the effect of steroid on VEGF/FLK1 signal pathway in ONFH rats.

In the present study, the steroid-induced osteonecrosis model was established in rats by three injections of high-dose MPSL as previously reported [23, 24]. The histopathology of osteonecrosis generated in this model was characterized by empty lacunae accompanied by surrounding narrow cell necrosis and occupation of adipocytes, which was similar to osteonecrosis in steroid-treated patients. However, there are some differences in the osteonecrosis observed in rats as compared to humans. For example, osteonecrosis often leads to femoral head collapse in humans but not in rats, as the epiphyseal line of the femur is permanent in adult rats but not in adult humans [30]. In the present study, the incidence of osteonecrosis (in the MPSL group) was 92%, and no rats died during the experimental period, indicating that this rat model is safe and effective. Thus, this model is useful for steroid induced osteonecrosis studies.

In our study, TMP treatment could significantly reduce the ration of empty lacunae and the area of bone marrow occupied by adipocytes, suggesting the improvement on necrosis of femoral head by TMP administration. Apart from the area of bone marrow occupied by adipocytes, TMP also decreased the serum markers of adipogenesis in steroid-induced ONFH rats, demonstrating the global effect of TMP in both systemic and local lipid metabolism in rats. In addition, the TMP treatment resulted in the decreased bone destruction in the femoral head, and these changes were associated with higher BV/TV and Tb.N. TMP also improved the trabecular microarchitecture, partially by restoring trabecular connectivity through increasing Tb.Th while reducing Tb.Sp, which is consistent with the increase in BMD, suggesting that TMP could prevent the loss of bone mass induced by the excessive steroid treatment.

Since the impeded blood flow through the femoral head is implicated in the aetiopathogenesis of steroid-induced ONFH, our study utilized a novel technique, using micro-CT-based microangiography, to visualize and quantify new blood vessel formation and vascularization in rat femoral head. Recent studies have demonstrated that this technique is quantitative and effective for assessing vascularization [26, 27]. Consistent with the improvement of the TMP treatment on the microstructure of the trabecular bone and BMD, the TMP-treated groups showed a significant increase in blood vessel volume, vessel surface, percentage of vessel volume, and vessel thickness, suggesting this administration may also increase vascularization of the femoral heads in rat model. These findings imply that TMP treatment might produce an environment conducive to prevention bone destruction by generating a blood supply.

The local blood supply and vascularization of the femoral head are generally considered to be involved in the occurrence of ONFH [31]. Glucocorticoids could decrease bone angiogenesis by suppression VEGF production of osteoblasts and osteocytes as well as by interference with VEGF action [3, 32]. Surgical procedures that aim to enhance revascularization of the necrotic area by applying angiogenic growth factor (mainly VEGF) have shown viable results, demonstrating that VEGF and blood supply may play a role in the development of osteonecrosis [33, 34]. VEGF, as an important mediator of angiogenesis, stimulates endothelial cells proliferation, promotes neovascularization, increases vascular permeability [35, 36], and plays a critical role in vascularization and vessel function [33]. During the process of angiogenesis, VEGF/FLK1 is the key signaling system to regulate the proliferation and migration of endothelial cells forming the basis of any vessel. In the current study, we found the downregulation of VEGF and FLK1 protein and gene expression in the sera and the necrotic femoral head, which were reversed by the treatment of TMP. In this respect, we speculate that TMP might prevent the effect of steroid on VEGF/FLK1 signal pathway which has been considered as a potential target for enhancing revascularization in steroid-induced ONFH patients. Furthermore, because VEGF is essential for bone formation [37], it may act as a key regulator that couples angiogenesis, bone formation, and repair of necrotic subchondral bone in femoral heads treated with TMP.

In conclusion, our data offer the convincing evidence for the first time that TMP may prevent steroid-induced ONFH and also enhance femoral head neovascularization by inhibiting the effect of steroid on VEGF/FLK1 signal pathway. These findings highlight the improvement of TMP on the blood supply and neovascularization during the progression of steroid-induced ONFH.
